# Ginsenoside Rb1 Ameliorated Bavachin-Induced Renal Fibrosis via Suppressing Bip/eIF2α/CHOP Signaling-Mediated EMT

**DOI:** 10.3389/fphar.2022.872474

**Published:** 2022-07-08

**Authors:** Yu-Hao Ni, Hui-Fang Deng, Lei Zhou, Cong-Shu Huang, Ning-Ning Wang, Lan-Xin Yue, Gao-Fu Li, Hui-Jing Yu, Wei Zhou, Yue Gao

**Affiliations:** ^1^ Department of Pharmaceutical Sciences, Beijing Institute of Radiation Medicine, Beijing, China; ^2^ School of Traditional Chinese Medicine, Guangdong Pharmaceutical University, Guangzhou, China; ^3^ Tianjin University of Traditional Chinese Medicine, Tianjin, China; ^4^ School of Pharmacy, Guangdong Pharmaceutical University, Guangzhou, China

**Keywords:** bavachin, ER stress, EMT, renal fibrosis, ginsenoside Rb1

## Abstract

The nephrotoxicity of *Fructus Psoraleae*, an effective traditional Chinese medicine for vitiligo treatment, has been reported. As one of the main toxic components in *Fructus Psoraleae*, bavachin (BV) was considered to be related to *Fructus Psoraleae*-caused adverse outcomes, but the direct evidence and molecular mechanism underlying BV-induced nephrotoxicity are not well elucidated. Therefore, this study was designed to confirm whether BV would cause toxic effects on the kidney and explore the possible mode of action. Our results demonstrated that days’ treatment with 0.5 μM BV indeed caused obvious renal fibrosis in the zebrafish kidney. The obvious E- to N-cadherin switch and the expressions of proteins promoting epithelial–mesenchymal transition (EMT) were observed in BV-treated human renal tubular epithelial and zebrafish kidneys. In addition, elevated reactive oxygen species (ROS) levels and Bip/eIF2α/CHOP-mediated endoplasmic reticulum (ER) stress and the unfolded protein response (UPR) were caused by BV, both of which could be reversed by ROS scavenger N-acetyl-L-cysteine (NAC). Also, blocking ER stress-caused cytoplasmic Ca^2+^ overload with 4-PBA notably alleviated BV-induced alterations in key molecular events related to EMT and renal fibrosis. Furthermore, of the natural compounds subjected to screening, ginsenoside Rb1 significantly downregulated BV-induced ER stress by inhibiting ROS generation and following the activation of Bip/eIF2α/CHOP signaling in HK2 cells. Subsequently, BV-triggered EMT and renal fibrosis were both ameliorated by ginsenoside Rb1. In summary, our findings suggested that BV-induced ROS promoted the appearance of EMT and renal fibrosis mainly via Bip/eIF2α/CHOP-mediated ER stress. This ER stress-related toxic pathway might be a potential intervention target for BV-caused renal fibrosis, and ginsenoside Rb1 would be a promising drug against BV- or *Fructus Psoraleae*-induced nephrotoxicity.

## 1 Introduction

Traditional Chinese medicine (TCM), effective alternative therapy for various diseases, has been widely used in China and other Asian countries for thousands of years (X. Liu et al., 2019). However, the complex components and unclear potential toxicity of TCM seriously hindered their clinical application (R. Liu et al., 2020; Zhong, Menon, Deng, Chen, & He, 2015). In recent years, several studies have demonstrated that some TCM could lead to chronic kidney disease (CKD) and renal fibrosis, which has been considered the final pathway for the development of CKD and was characterized by tubular epithelial–mesenchymal transition (EMT), interstitial myofibroblast activation, excessive extracellular matrix deposition, and fibrotic tissue remodeling.

*Fructus Psoraleae* (Buguzhi), the dry and mature fruit of plant *Cullen corylifolium* (L.) Medik. (Fabaceae) (syn. of *Psoralea corylifolia* L.), is a well-known TCM for external as well as internal treatment of various diseases, for instance, alopecia, inflammation, leukoderma, leprosy, psoriasis, and eczema from ancient times (Alam, Khan, & Asad, 2018). Regardless of its rich pharmacological activities, the adverse effects of *Fructus Psoraleae* have also been reported in recent years. For instance, *Fructus Psoraleae* decoction caused hepatotoxicity and nephrotoxicity in rats after 7 days of administration (A. Li et al., 2016). *In vitro* studies showed that *Fructus Psoraleae* could induce toxicity to human renal tubular epithelial cells by damaging cell membrane, inducing cell apoptosis, inhibiting intracellular DNA synthesis, blocking cell mitosis, and inhibiting cell proliferation (F. Jiang, Zhou, Wang, & Zhang, 2010). But the exact toxic component and molecular mechanism underlying *Fructus Psoraleae*-induced nephrotoxicity were not fully understood. Bakuchiol, as the main constituent of *Fructus Psoraleae*, was found to have a toxic effect on mouse liver and kidney at high doses (Hu et al., 2018; Z. J. Li et al., 2017; Y. Zhang, Liu, Wu, & Wang, 1981). However, our previous study demonstrated that bavachin (BV), another newly discovered toxic substance in *Fructus Psoraleae*, could cause obvious hepatotoxicity at very low doses, implying BV might be one of the main toxic components of *Fructus Psoraleae*-induced nephrotoxicity (Y. Yang et al., 2018).

EMT refers to the switching of cells from an epithelial state to a mesenchymal fibroblast state, accompanied by decreased intercellular adhesion and increased motility. There are three different subtypes of EMT, including type I EMT, type II EMT, and type III EMT. Type II EMT associated with fibrosis has commonly been considered the main fibrogenic process in renal fibrosis. More than 30% of the novel fibroblasts originate from local EMT (X. Liu et al., 2019). Furthermore, recent studies have identified EMT as a key determinant of the progression from adaptive to maladaptive repair in renal fibrosis (Lovisa et al., 2015; Qi et al., 2021). In general, the initiation of EMT aims to prevent epithelial stress response, but overactivated EMT significantly affects renal tubular epithelial cells uptake and secretion functions, induces G2 cell cycle arrest, and impairs tissue repair (Lovisa et al., 2015). Furthermore, EMT could cause interstitial fibrosis, inflammation, and immune recruitment by altering the epithelial secretome profile (Vincent & Fuxe, 2017; Wang et al., 2020). But so far, no direct evidence has focused on the relationship between BV and EMT.

Mechanistically, excessive oxidative stress and/or inflammatory reaction-induced EMT have been supposed to play critical roles in TCM-induced renal fibrosis (Cybulsky, 2017; Ji et al., 2019; Zhou et al., 2020a). Usually, the endoplasmic reticulum senses cellular stress and subsequently restores cellular homeostasis by triggering the ER stress and downstream unfolded protein response (UPR) (Cybulsky, 2017). In our previous study, we confirmed that BV-triggered ER stress participated in *Fructus Psoraleae*-induced hepatotoxicity (Yang et al., 2021). The latest articles showed that enhanced ER stress promoted EMT in human lens and lung epithelial cells (D. Liu et al., 2018; Zhou et al., 2020b). Also, there was crosstalk between the UPR and EMT under some conditions (Zhao, Zhang, Sun, & Brasier, 2021). Hence, we speculated that BV-triggered ER stress might contribute to *Fructus Psoraleae*-induced nephrotoxicity via EMT-mediated renal fibrosis.

In this present study, we first confirmed whether repeated BV treatment could induce renal fibrosis, ER stress, and EMT *in vivo* and *in vitro* and elucidated the possible molecular mechanism involved in BV-induced renal fibrosis, aiming to provide a specific intervention target for *Fructus Psoraleae*-caused nephrotoxicity.

## 2 Materials and Methods

### 2.1 Cell Culture and Treatments

Human proximal tubular epithelial cell line (HK2) cells were cultured in DMEM medium (Thermo Fisher Scientific, Waltham, MA, United States) supplemented with 10% fetal bovine serum (FBS, Sijiqing), 100 U/mL penicillin, and 100 μg/ml streptomycin (FG101-01, Transgene, China) and maintained in an incubator with 5% CO_2_ at 37°C. Cells were passaged at 80% confluence using 0.25% trypsin-EDTA, and the medium was renewed every 2 days.

### 2.2 Protein Extraction and Western Blot

Equal amounts of protein from HK2 cells were loaded onto SDS-PAGE gels. After electrophoresis and transference, membranes were blocked with 5% nonfat dry milk in TBS-T and incubated overnight at 4°C with different primary antibodies. Subsequently, membranes were incubated with a secondary antibody anti-mouse IgG (CST, 1:3,000) or anti-rabbit IgG (CST, 1:3,000). Protein bands were detected by LAS-300 after treatment with enhanced chemiluminescence (ECL) according to the manufacturer’s instructions (WBKLS0500, Millipore). Band intensity of each protein was quantified using ImageJ software. Results were normalized to GAPDH for total proteins to control. Information for all indicated antibodies used in our study can be found in Table 1.

**TABLE 1:** Information for all indicated antibodies used in western blots assay.

	Name	Company	Dilution rate
1	α-SMA	19,245, Cell Signaling Technology, United States	1:1,000
2	Notch1	3,608, Cell Signaling Technology, United States	1:1,000
3	c-Myc	18,583, Cell Signaling Technology, United States	1:1,000
4	Hes1	11,988, Cell Signaling Technology, United States	1:1,000
5	NICD	4,147, Cell Signaling Technology, United States	1:1,000
6	GAPDH	200,310-4F11, ZEN BIO, China	1:1,000
7	E-Cadherin	66009-1-Ig, Proteintech, United States	1:1,000
8	N-Cadherin	13,116, Cell Signaling Technology, United States	1:1,000
9	Vimentin	5,741, Cell Signaling Technology, United States	1:1,000
10	ATP1A1	ab7671, Cell Signaling Technology, United States	1:1,000
11	AQP1	ab168387, Abcam, United States	1:1,000
12	SLC22A6	ab135924, Abcam, United States	1:1,000
13	ZEB1	70,512, Cell Signaling Technology, United States	1:1,000
14	Claudin-1	13,995, Cell Signaling Technology, United States	1:1,000
15	Slug	9,585, Cell Signaling Technology, United States	1:1,000
16	ZO-1	13,663, Cell Signaling Technology, United States	1:1,000
17	Bip	3,177, Cell Signaling Technology, United States	1:1,000
18	ATF6	500,202, ZEN BIO, China	1:1,000
19	p-perk	340,846, ZEN BIO, China	1:1,000
20	ATF-4	11,815, Cell Signaling Technology, United States	1:1,000
21	IRE1α	3,294, Cell Signaling Technology, United States	1:1,000
22	XBP-1s	12,782, Cell Signaling Technology, United States	1:1,000
23	CHOP	15204-1-AP, Proteintech, United States	1:1,000
24	Anti-Rb IgG	ab6721, Abcam, United States	1:3,000
25	Anti-Ms IgG	ab6789, Abcam, United States	1:3,000
26	p-eIF2α	310,073, ZEN BIO, China	1:1,000
28	Smad3	385,743, ZEN BIO, China	1:1,000

### 2.3 Detection of the Level of Cytoplasmic Calcium (Ca^2+^)

Cytosolic calcium ions (Ca^2+^) in cells were monitored using Fluo 4-AM (F312, Dojindo, Japan). The cells were seeded in glass culture dishes at a density of 2.0 × 10^4^/ml. After 24 h treatment with BV (20 μM) with or without NAC (5 mM) or ginsenoside Rb1 (40 μM), cells were stained with 1 μM Fluo 4-AM for 10 min according to the manufacturer’s instructions. The extra dye was discarded by a three-time wash with HBSS. The concentration of cytoplasmic Ca^2+^ was detected by living cell imaging. The excitation and emission wavelengths were 494 nm and 516 nm respectively.

### 2.3 Detection of the Level of Cytoplasmic Reactive Oxygen Species

Cytosolic ROS in cells were detected using MitoSOX (M36008, Invitrogen, America). The cells were seeded in glass culture dishes at a density of 2.0 × 10^4^/ml. After 24 h treatment with BV (20 μM) with or without NAC (5 mM) or ginsenoside Rb1 (40 μM), cells were stained with 5 μM MitoSOX for 20 min according to the manufacturer’s instructions. The extra dye was discarded by a three-time wash with PBS. The final image was captured by living cell imaging.

### 2.4 Drug Screening

A total of 6 compounds, including ginsenoside Rb1, ginsenoside Re, ferulic acid, digitoflavone, salidroside, and liquiritin, were dissolved in DMSO or distilled water at 100 mM, 100 mM, 1 M, 1 mM, 100 mM, and 50 mM, respectively. Drugs were then diluted in DMEM (Life Technologies, Thermo Fisher Scientific) supplemented with 100 U/ml penicillin and 100 μg/ml streptomycin (PS; Life Technologies, Thermo Fisher Scientific) to the final concentration. HK2 cells treated with drugs were then incubated at 37°C and 5% CO_2_ in a humidified incubator in 96-well plates for final concentrations of 40 μM, 10 μM, 500 μM, 1 μM, 100 μM, and 50 μM, respectively, in a final volume of 100 μl DMEM for 24 h. Cytosolic calcium ions (Ca^2+^) in cells were detected as mentioned before.

### 2.5 Zebrafish Husbandry and Treatments

A 21-day exposure of DMSO (0.5 μM), bavachin (0.5 μM), and ginsenoside Rb1 (2 μM) with bavachin (0.5 μM) on adult zebrafish was performed, each containing 10 fish in triplicates with the solutions being renewed daily. The body length and wet weight of all living individuals were measured at the beginning and end of the test. The fish were fed daily with dry food (equivalent to 3% of body weight, Porpoise Aquarium Co., Ltd.). All experiments involving animals were approved by the Ethics Committee of the Beijing Institute of Radiation Medicine (approval no. IACUC-DWZX-2022–602).

### 2.6 Histological Studies

Fixed kidney tissues of zebrafish were embedded in paraffin and cut into 5 µm sections. Masson’s trichrome staining was performed to detect connective tissue according to the manufacturer’s instructions. Blue coloration was indicative of collagen deposition in Masson’s trichrome.

### 2.7 *In Situ* Hybridization

*In situ* hybridization was carried out according to Barth and Wilson (Barth & Wilson, 1995). After exposure to 0.5 μM BV media for 21 days, the fish kidney tissues were fixed with *in situ* hybrid fixative solution (Servicebio) overnight at room temperature. Sections for *in situ* hybridization were harvested and treated with 10 μg/ml proteinase K (sigma) in PBS at 37°C for 30 min. After a 5 min fixation with 4% paraformaldehyde (PFA) and 2 × 5 min rinses with PBS, sections were incubated with hybridization buffer (Hyb [−] and Hyb [+]) containing 2 μl probes each milliliter at 43°C for 1 h and 12 h, respectively. Following hybridization, sections were washed with 2x SSC, 1x SSC, 0.5x SSC, and 0.1x SSC sequentially and incubated with MAB for 15 min each step. Then, sections were incubated with 1:3,000 diluted anti-DIG antibody at 37°C for 4 h followed by blocking with a blocking buffer containing 2% blocking reagent (Roche, Germany) for 30 min at room temperature. 1x PBS, PTW, and NTMT buffer (100 mM NaCl, 50 mM MgCl_2_, 100 mM Tris-HCl, pH 9.5) were needed to wash sections, respectively, for 5 min twice, 5 min twice, 10 min twice. The color reaction was carried out with incubation in BM-purple substrate at room temperature for 30 min. The last 5 min-wash with PBS was needed before 30 min-fixation with 4% PFA.

### 2.8 Statistical Analysis

All values are expressed as means ± SEM and analyzed using the GraphPad Prism 7 (GraphPad Software, Inc., La Jolla, CA). One-way ANOVA, followed by Tukey’s test and two-way ANOVA, was employed to analyze significance when comparing multiple independent groups. In each experiment, *N* represents the number of independent experiments (*in vitro*) and the number of zebrafish (*in vivo*). The data were considered statistically significant at *p* < 0.05.

## 3 Results

### 3.1 Long-Term Treatment With a Low Dose of Bavachin-Induced Renal Fibrosis in Zebrafish

**FIGURE 1:**
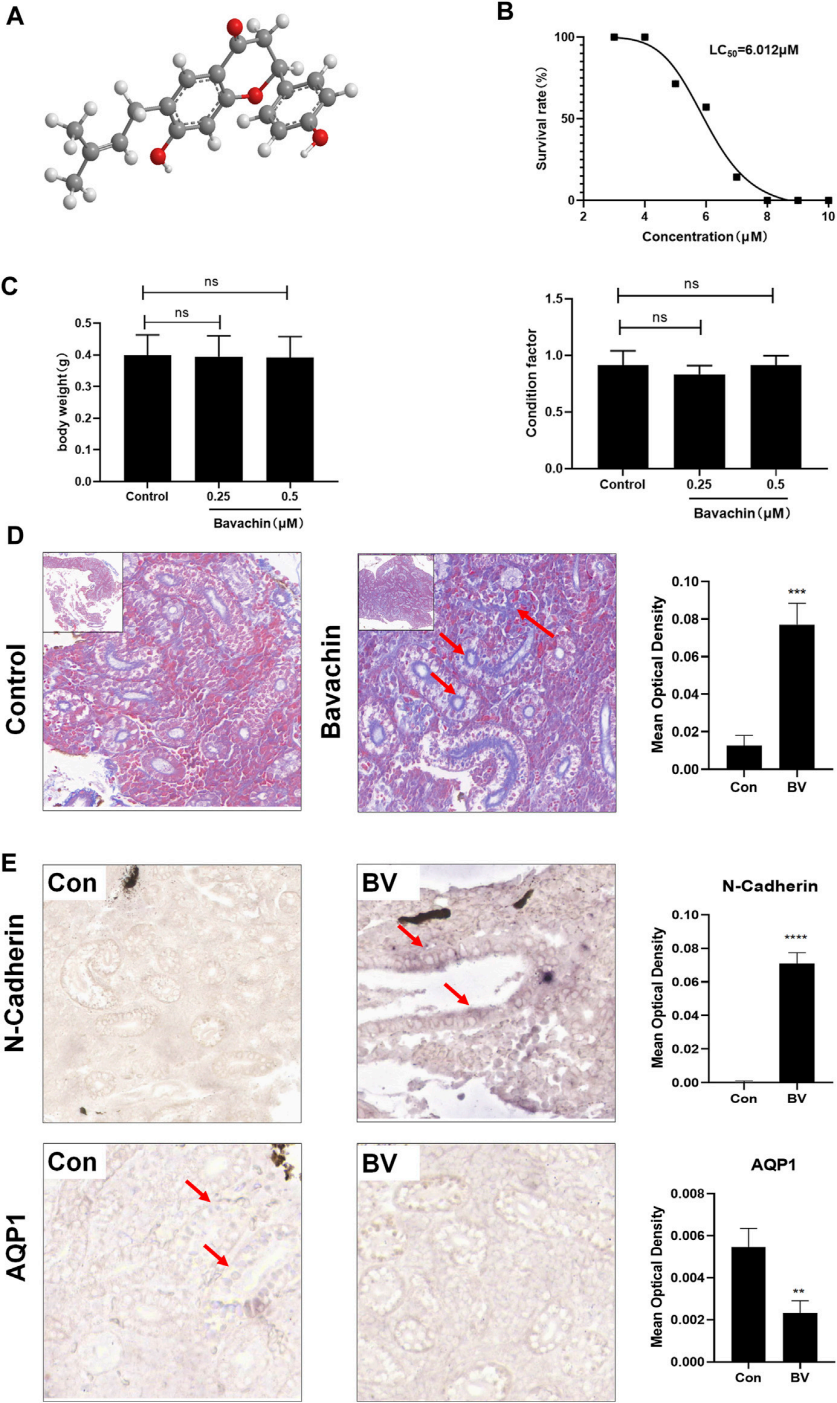
Long-term treatment with a low dose of BV-induced renal fibrosis in zebrafish. **(A)** Chemical structure of BV. **(B)** LC_50_ of BV on zebrafish after 4 days of treatment was calculated. **(C)** Body weight and condition factor of zebrafish after 21 days BV treatment (n = 30). **(D)** Masson trichrome staining of zebrafish kidney treated with solvent or BV, respectively. **(E)** The contents of N-cadherin and AQP1 in zebrafish kidneys were detected by *in situ* hybridization. ns means no significant difference. **p < 0.01, ***p < 0.001, and ****p < 0.0001 vs. the control groups.

In order to determine the dose that did not induce any acute toxicity, we determined the LC_50_ value of BV (6.012 μM) through acute toxicity experiments (Figure 1B). Next, to evaluate the toxic effects of BV on kidneys, zebrafish were repeatedly treated with 0.5 μM of BV for 21 days and repeated treatment with BV did not alter the body weight and condition factor of zebrafish (Figure 1C). Then, the zebrafish renal tissue was determined morphologically by Masson trichrome staining, demonstrating that massive collagen deposition was observed in renal tubulointerstitium and the tubular compartment and diffusely expanded among whole samples, while no fibrotic lesion was found in solvent-treated zebrafish kidneys (Figure 1D). Moreover, the result of *in situ* hybridization showed that the transcription level of epithelial marker AQP1 decreased significantly after 21-day BV administration (Figure 1E), but the content of N-cadherin, one of the markers of EMT, dramatically increased, indicating the possible occurrence of E- to N-cadherin switch in zebrafish renal tubular epithelium. Taken together, these results indicate that long-term treatment with BV could induce the loss of normal tissue structure in the renal tubular epithelium and obvious renal fibrosis in zebrafish.

### 3.2 Bavachin Caused Perturbations in Fibrosis and Epithelial–Mesenchymal Transition-Relevant Signaling Pathways *In Vitro*

**FIGURE 2:**
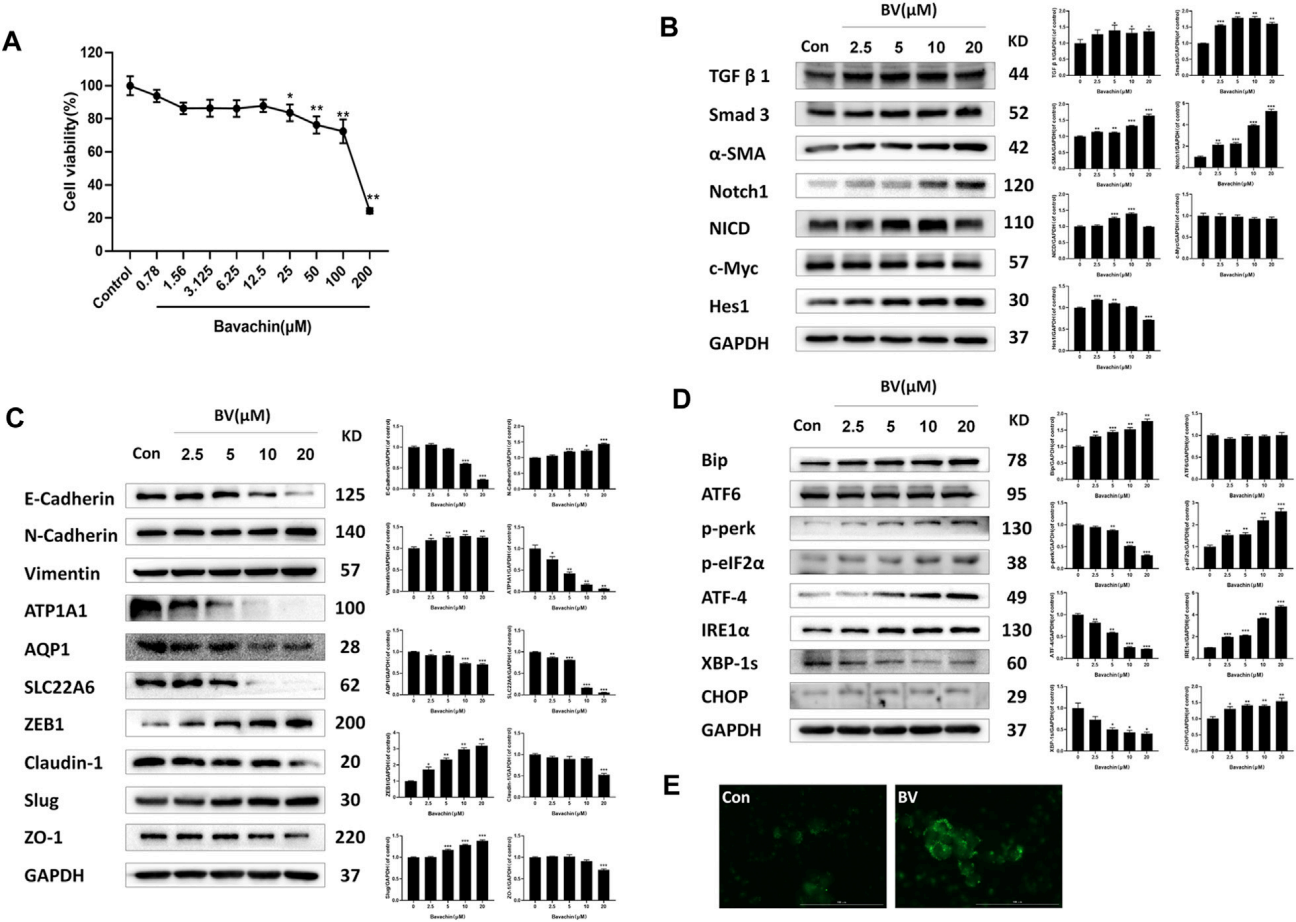
BV caused perturbations in ER stress-, fibrosis- and EMT-relevant signaling pathways *in vitro*. **(A)** Cell viability of HK2 cells after 24 h BV treatment was detected by CCK-8 assay. The expressions of indicated proteins involved in fibrosis **(B)** and EMT **(C)** in HK2 cells were measured using western blot. **(D)** The expressions of ER stress and UPR-associated proteins in BV-treated cells. **(E)** The intracellular concentration of Ca^2+^ was demonstrated using Fluo 4-AM (scale bar: 100 μm). *p < 0.05, **p < 0.01, and ***p < 0.001 vs. the control groups.

Furthermore, we investigated the molecular mechanisms underlying BV-induced nephrotoxicity *in vitro*. First, we exposed HK2 cells to various concentrations (0–200 μM) of BV for 24 h to determine the cytotoxicity of BV on human renal tubular epithelial cells. As shown in Figure 2A, cell viability was reduced significantly by BV in a dose-dependent manner. It was found that 25 μM of BV decreased HK2 cell viability to 82.10 ± 2.43%. We then detected the expressions of fibrosis-associated proteins in HK2 cells by western blot assay. As shown in Figure 2B, BV obviously increased the expressions of TGFβ1, Smad3, α-SMA, Notch1, NICD, c-Myc, and Hes1 in a dose-dependent manner. Then, we assessed the contents of EMT-related proteins in HK2 cells after 24 h treatment with BV and found a remarkable E-to N-cadherin switch in BV-treated renal tubular epithelial cells (Figure 2C). Compared to the control group, BV dose-dependently upregulated the contents of vimentin, ZEB1, and slug, but reduced claudin-1 and ZO-1 expression in HK2 cells. In addition, BV treatment caused the loss of specific functional proteins, including ATP1A1, AQP1, and SLC22A6, in human renal tubule epithelia (Figure 2C).

### 3.3 Bavachin Activated Endoplasmic Reticulum Stress Following Cellular Ca^2+^ Overloading via Elevating Reactive Oxygen Species

**FIGURE 3:**
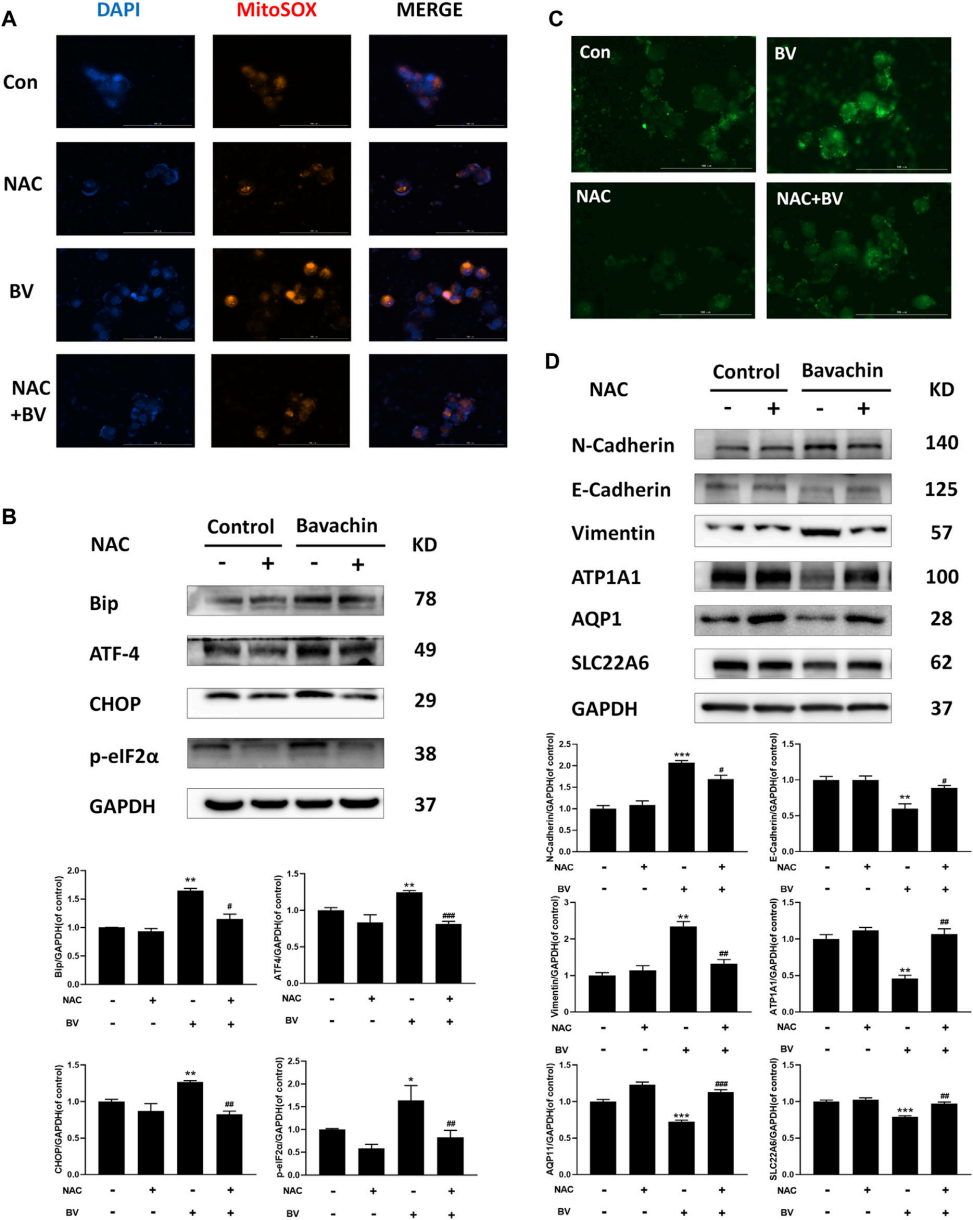
Elevating ROS contributed to BV-triggered ER stress and EMT. **(A)** Intracellular ROS levels in HK2 cells induced by BV with or without NAC treatment (scale bar: 100 μm). After NAC treatment, ER stress- **(B)**, EMT- **(D)** related proteins, and cellular Ca^2+^ concentration **(C)** were detected with western blot and Fluo 4-AM, respectively. *p < 0.05, **p < 0.01, and ***p < 0.001 vs. the control groups. ^**#**^ *p* < 0.05, ^**##**^ *p* < 0.01, and ^**###**^ *p* < 0.001 vs. the BV-treated groups.

Given that ER stress played a crucial role in BV-induced hepatotoxicity, we next explored whether ER stress existed in BV-treated human renal tubular epithelial cells. As shown in Figure 2D, compared with the control group, the expressions of Bip, p-perk, p-eIF2α, ATF-4, IRE1α, and CHOP were markedly increased in a dose-dependent manner, but that of XBP1s was obviously decreased in the BV-treated groups. Also, there were no significant changes in ATF6 content among the BV-treated groups and the control group (Figure 2D). Moreover, the HK2 cells were stained with Fluo-4 AM to show the change in intracellular Ca^2+^ content after BV treatment. Our data showed obvious enhancement in cytoplasmic Ca^2+^ loading in the BV-treated group (20 μM) (Figure 2E). Furthermore, BV increased the intracellular ROS levels in HK2 cells, and cotreatment with NAC (5 mM), a scavenger of ROS, alleviated BV-elevated intracellular ROS (Figure 3A). Also, cotreatment with NAC significantly reversed BV-increased the expressions of Bip, ATF4, p-eIF2α, and CHOP (Figure 3B) and restored cytoplasmic Ca^2+^ concentration (Figure 3C). These results showed that BV could induce an increase in ROS and Bip/eIF2α/CHOP signaling pathway-mediated ER stress, which eventually caused Ca^2+^ release from the ER and Ca^2+^ overloading in the cytoplasm.

### 3.4 Inhibition of Reactive Oxygen Species–Mediated Endoplasmic Reticulum Stress Reversed Bavachin-Triggered Epithelial–Mesenchymal Transition and Profibrotic Signaling

**FIGURE 4:**
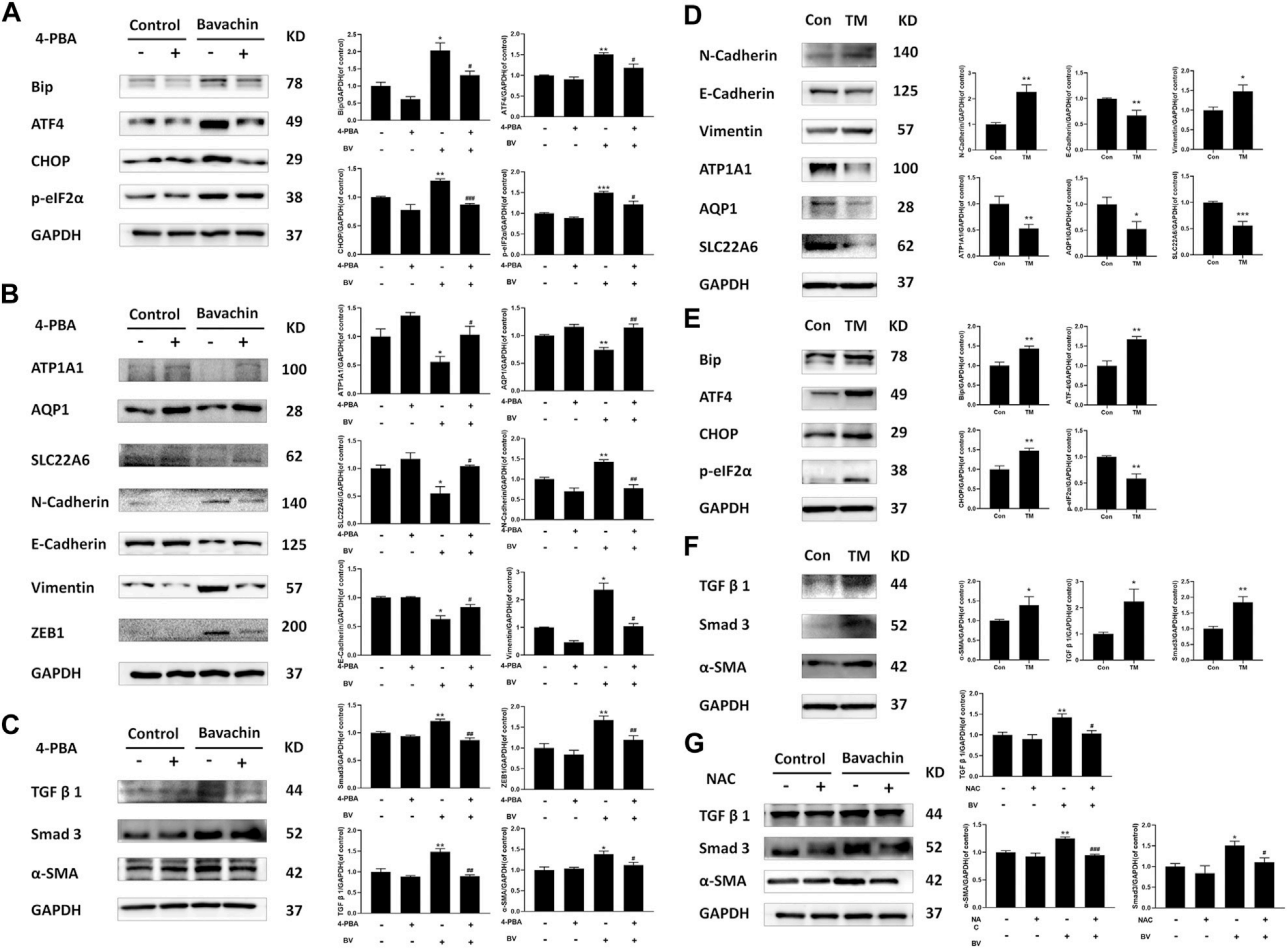
Inhibition of ROS-mediated ER stress reversed BV-triggered EMT and profibrotic signaling. After 24 h BV treatment with/without 4-PBA co-treatment, the expressions of ER stress **(A)**, EMT **(B),** and fibrosis-related **(C)** proteins were examined by western blot. **(D–F)**The expressions of indicated proteins in TM-treated HK2 cells. **(G)** BV-induced profibrotic signaling in NAC cotreated HK2 cells was detected. *p < 0.05, **p < 0.01, and ***p < 0.001 vs. the control groups. ^**#**^ *p* < 0.05, ^**##**^ *p* < 0.01, and ^**###**^ *p* < 0.001 vs. the BV-treated groups.

To elucidate the possible role of ER stress in BV-triggered EMT and fibrosis, we detected the expressions of ER stress-related proteins after 24 h BV treatment with/without 4-PBA (an inhibitor of ER stress, 2 mM). Obviously, 4-PBA cotreatment indeed inhibited the activation of ER stress (Figure 4A) and also prevented BV-induced E- to N-cadherin switch and the proteins involved in pro-EMT or renal fibrosis, while restoring the expressions of AQP1, ATP1A1, and SLC22A6 in human renal tubule epithelia (Figure 4B). As a specific agonist of ER stress, TM alone also enhanced Bip/eIF2α/CHOP pathway-mediated ER stress and promoted the expressions of the indicated proteins associated with EMT and fibrosis (Figures 4D–F). Interestingly, NAC cotreatment also decreased the levels of pro-EMT (N-Cadherin, Vimentin) (Figure 3D) and fibrosis-related signaling (TGFβ1, Smad3, and α-SMA) (Figure 4G) in BV-treated HK2 cells. Like 4-PBA, NAC could preserve the structural markers of the renal tubular epithelium (AQP1, ATP1A1, and SLC22A6) in BV-treated HK2 cells (Figure 3D). Altogether, these results verified that ROS-activated ER stress participated in BV-induced EMT and renal fibrosis.

### 3.5 Ginsenoside Rb1 Alleviated Bavachin-Induced Epithelial–Mesenchymal Transition and Renal Fibrosis by Downregulating Reactive Oxygen Species–Mediated Endoplasmic Reticulum Stress

**FIGURE 5:**
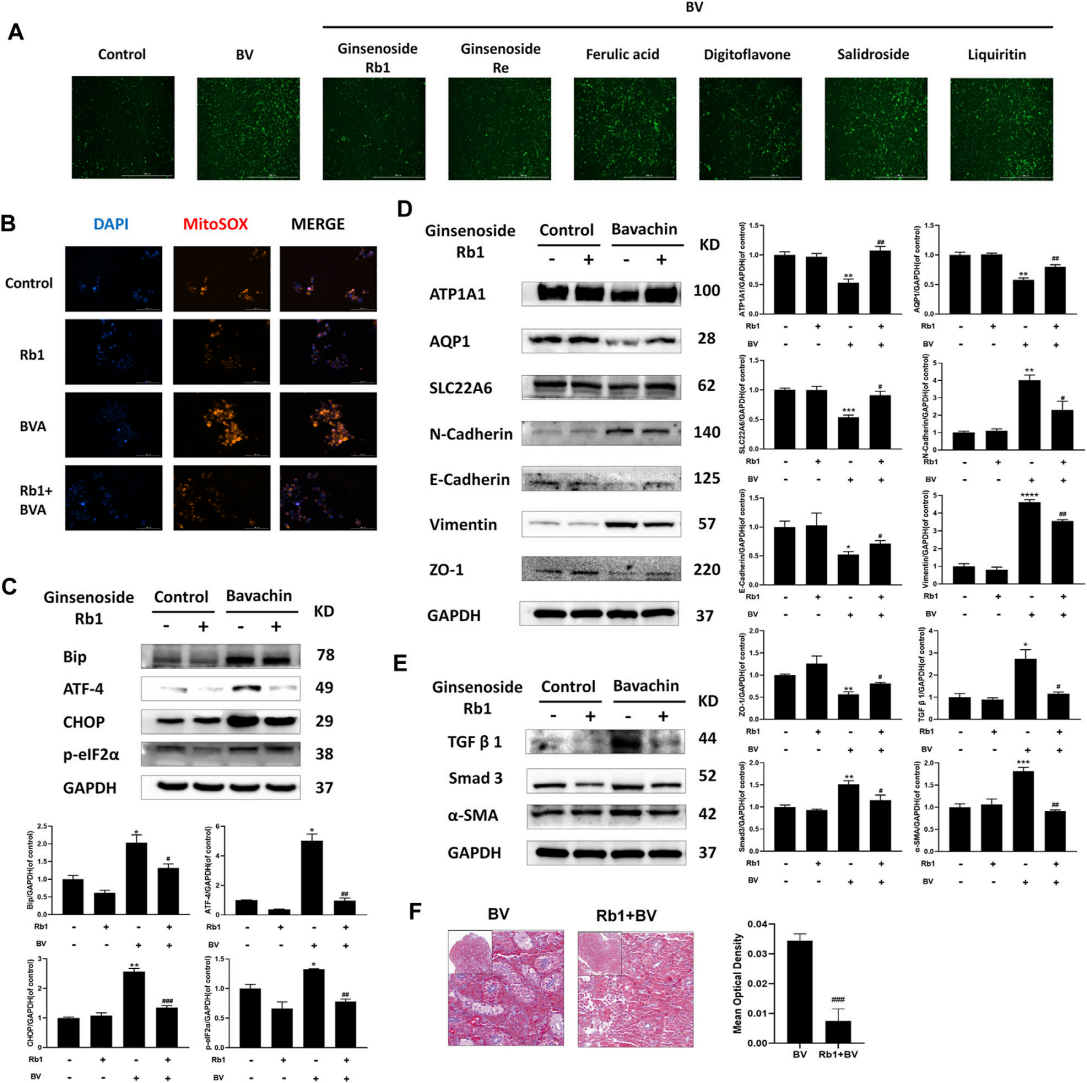
Ginsenoside Rb1 alleviated BV-induced EMT and renal fibrosis by downregulating ROS-mediated ER stress. **(A)** Screening for the drug inhibiting intracellular Ca^2+^ release. **(B)** After ginsenoside Rb1 cotreatment, the ROS levels in each group were detected (scale bar: 100 μm). The indicated proteins participating in ER stress **(C)**, EMT **(D)**, and fibrosis **(E)** in HK2 cells cotreated BV with/without ginsenoside Rb1. **(F)** Masson trichrome staining of zebrafish kidney cotreated BV with/without ginsenoside Rb1. *p < 0.05, **p < 0.01, and ***p < 0.001 vs. the control groups. ^**#**^ *p* < 0.05, ^**##**^ *p* < 0.01, and ^**###**^ *p* < 0.001 vs. the BV-treated groups.

Since ROS-mediated ER stress contributed to BV-induced EMT and renal fibrosis, we next performed high throughput screening (HTS) to find potential drugs antagonizing BV-generated nephrotoxicity using fluorescence calcium, which would indicate the activation of BV-triggered ER stress. Ginsenoside Rb1, ginsenoside Re, ferulic acid, digitoflavone, salidroside, and liquiritin were subjected to HTS. The results preliminarily demonstrated that ginsenoside Rb1, ginsenoside Re, and ferulic acid could ameliorate BV-elevated cytoplasmic Ca^2+^ overloading in HK2 cells (Figure 5A). Also, ginsenoside Rb1 (40 μM) could reduce the levels of ROS in both normal and BV-treated cells (Figure 5B). In the subsequent experiment, only cotreatment with ginsenoside Rb1 could reduce BV-upregulated expressions of Bip, following ATF4, p-eIF2α, and CHOP (Figure 5C). *In vitro* experiments showed that elevated TGFβ1/Smad3 signaling, as well as the molecules involved in the progress of BV-triggered EMT, were partly abrogated by ginsenoside Rb1 (Figures 5D,E). In addition, cotreatment with ginsenoside Rb1 completely restored the structural markers of the renal tubular epithelium (AQP1, ATP1A1, and SLC22A6) in BV-treated HK2 cells (Figure 5D). Furthermore, ginsenoside Rb1 significantly alleviated BV-induced tubule fibrosis in zebrafish kidneys (Figure 5F). Hence, our data confirmed a protective role of ginsenoside Rb1 in the BV-caused EMT and renal fibrosis via inhibiting ROS-mediated ER stress.

## 4 Discussion

Since thousands of years ago, *Fructus Psoraleae* has been widely used clinically for various disease therapy, for instance, spermatorrhea, vitiligo, and lumbago. Recently, increasing studies have reported *Fructus Psoraleae* could cause obvious toxic effects on the liver and kidney (Nam et al., 2005; Y. Zhang et al., 1981). But compared with hepatotoxicity, little research focused on *Fructus Psoraleae*-induced nephrotoxicity and its underlying mechanism. In addition, our previous studies found that being one of the most important components, BV could induce evident adverse outcomes at a very low dose and was presumed to be relevant to *Fructus Psoraleae*-induced nephrotoxicity (Jia-yan et al., 2021; Y. Yang et al., 2018). In this study, we first confirmed that long-term treatment with BV could induce apparent EMT and renal fibrosis in zebrafish kidneys. Mechanistically, BV treatment led to ROS generation and the activation of the Bip/eIF2α/CHOP signaling pathway and subsequently caused ER stress, UPR, and cytoplasmic Ca^2+^ overloading, both of which in turn regulated the expressions of classical signals involved in EMT and fibrosis, ultimately resulting in renal fibrosis in renal tubular epithelial cells. Furthermore, for the first time, ginsenoside Rb1 was verified to function as a protective agent against BV-induced EMT and renal fibrosis *in vivo* and *in vitro* through inhibiting ROS generation and Bip/eIF2α/CHOP toxicity pathway and following Ca^2+^ overload.

Renal fibrosis is considered the common final pathway of almost all nephropathies and is characterized by oxidative/inflammatory responses, fibroblast activation, excessive deposition of extracellular matrix, and fibrotic tissue remodeling (Nastase, Zeng-Brouwers, Wygrecka, & Schaefer, 2018). Of a wide range of insults, several Chinese herbs had been reported in relevant to drug-induced nephropathy, including *Aristolochiae manshuriensis* Kom. and *Fructus Psoraleae* (B. Yang et al., 2018). Different from the former, the exact toxic component and toxicity model of action involved in *Fructus Psoraleae*-induced renal injuries were not entirely elucidated. Consistent with previous studies and our earliest hypothesis, this study first identified that repeated treatment with low-dose of BV, one of the main toxic ingredients in *Fructus Psoraleae*, caused significant renal interstitial and tubular epithelial deposition of collagen fibers in zebrafish kidneys. The latest evidence showed that adverse effects on tubular epithelial cells acted as a driving force that initiated and promoted interstitial injuries and consequent fibrosis upon exposure and response to various insults (H. Li et al., 2019). Generally, AQP1, one of the water channel aquaporin isoforms highly expressed in the renal tubular epithelium, participates in the maintenance of body water and sodium homeostasis (J. Li et al., 2018; Su J. et al., 2020). N-cadherin, the adhesion marker of mesenchymal cells, expresses in fibrous, nervous, and musculoskeletal tissues, but does not largely exist in the epithelium (Noronha et al., 2021). Interestingly, our results presented an obvious reduction of AQP1 expression in the renal tubular epithelial cells of BV-treated zebrafish, indicating that long-term BV treatment induced an ongoing loss of normal structure-specific properties of tubular epithelium. Also, enhanced N-cadherin content was closely related to the acquisition of mesenchymal properties in the renal tubular epithelium, namely, EMT. Therefore, low-dose of BV-induced renal fibrosis could be EMT-dependent.

Moreover, *in vitro* results showed that cellular expressions of cadherins were dramatically disturbed in BV-treated HK2 cells. Apart from the enhancement of N-cadherin, the expression of E-cadherin, which regulated epithelial tissue integrity, was substantially inhibited by BV. This phenomenon was usually called E- to N-cadherin switch and is considered as the hallmark of EMT. Altered cadherin expression is accompanied by the suppression of epithelial tight junctions or zonula occludens-related proteins (ZO1 and Claudin1) and the increases in E-cadherin–related transcriptional repressors (ZEB1 and slug), together with confirmed BV-triggered EMT in human renal tubular epithelium. Mechanistically, many upstream signaling pathways, including TGF-β, Wnt, and Notch1, were well-documented to regulate the developmental EMT and renal fibrosis (Edeling, Ragi, Huang, Pavenstadt, & Susztak, 2016; J.; Su W. et al., 2020). Except for the Wnt/β-catenin signal, BV indeed dose-dependently upregulated canonical TGFβ1/Smad 3 and Notch1/NICD signaling pathways. Meanwhile, significant fibroblast activation in BV-treated tubular epithelium was evidenced by increasing the expressions of α-SMA and vimentin, both of which formed bundles of stress fibers and filament. However, BV caused distinct depletions in several tubular epithelial cells’ specific transporters, including AQP1, SLC22A6, and ATP1A1 (known as the Na^+^/K^+^-ATPase pump). Hence, we further demonstrated that BV treatment could promote EMT and fibroblast-like properties’ acquisition in renal tubular epithelial cells through TGFβ1 and Notch1-mediated fibrotic signaling responses, all of which eventually caused renal fibrosis.

Oxidative responses always conduce to toxic effects induced by xenobiotics, including BV. Our previous work reported that BV caused ROS generation and finally promoted obvious cytotoxicity *in vivo* and *in vitro* (Y. Yang et al., 2018). Notably, ROS could increase the expression of slug, which was the transcriptional repressor of E-cadherin, by increasing the phosphorylation of extracellular signal-regulated kinase (ERK) (Z. Zhang et al., 2020), then downregulated cellular E-cadherin content, and changed the epithelial architecture via relegating cell-cell adhesion (Noronha et al., 2021). Also, overwhelming ROS-induced oxidative damages could activate HIF-1α and NF-κB, both of which drove the occurrence and progression of EMT following fibrosis (Gloire & Piette, 2009). In addition, ER, an elegant organelle that controls protein synthesis, folding, and trafficking, was reported to be vulnerable to excessive ROS (Zeeshan, Lee, Kim, & Chae, 2016). Oxidative stress or damage caused disruption of ER function and resulted in the accumulation of newly synthesized misfolded protein in the ER, known as ER stress. Subsequently, enhanced ER chaperone (Bip) binding to misfolded proteins could lead to the release of ER stress sensors, including IRE1α, PERK, and ATF6, and activate the following UPR to restore homeostasis via ER-associated degradation (ERAD) system (Cybulsky, 2017). Overt ER stress and following UPR were confirmed to play a critical role in BV-induced hepatotoxicity. But unlike unselective activation of the three classical ER stress sensors in BV-treated hepatocytes, only the Bip/PERK/eIF2a/ATF4 signaling pathway mediated BV-induced ER stress and UPR in HK2 cells. Furthermore, as the executors of ER stress-initiated mitochondrial apoptosis, CHOP and Ca^2+^ signaling could cause a profibrotic microenvironment by promoting apoptosis (Song, Gou, & Zhang, 2016; Zhong et al., 2021). Also, elevated Bip expression had been found to participate in EMT-associated metastasis (Kopp, Larburu, Durairaj, Adams, & Ali, 2019). Not surprisingly, when we reduced the levels of ROS and cytoplasmic Ca^2+^, BV-induced EMT and profibrotic signaling were alleviated, respectively. Altogether, ROS-induced ER stress could be the key modulator of BV-caused EMT and renal fibrosis.

Ginseng, one of the favorable natural medicines which have a long history of clinical use in east Asia for thousands of years, has been found to possess antioxidative, anti-inflammation, and anti-fibrosis properities (Mancuso & Santangelo, 2017). As one of the main effective ingredients in ginseng, ginsenoside Rb1 has shown excellent therapeutic effects on nervous and cardiovascular system diseases (Lin et al., 2022). In terms of pharmacological mechanisms, ginsenoside Rb1 limited ROS generation by suppressing the activity of NADH dehydrogenase (L. Jiang et al., 2021). On the other hand, ginsenoside Rb1 had been verified to target multiple pathways, including Nrf2/ARE, STAT3, and TGFβ1/Smad3 signaling pathways, to regulate redox balance, inhibit apoptosis, and attenuate cardiac fibrosis (Zheng et al., 2017; Wu, Huang, Bell, & Yu, 2018; Peng et al., 2021). Like the aforementioned efficacy, ginsenoside Rb1 not only inhibited BV-induced ROS generation but also decreased overactivated ER stress following UPR and cytoplasmic Ca^2+^ release. In particular, BV-caused sustained activation of EMT progress and renal fibrosis were significantly ameliorated by ginsenoside Rb1 after long-term combination administration. Hence, ginsenoside Rb1 was proposed to be a valuable ingredient in the therapy of BV-caused renal fibrosis.

## 5 Conclusion

**FIGURE 6:**
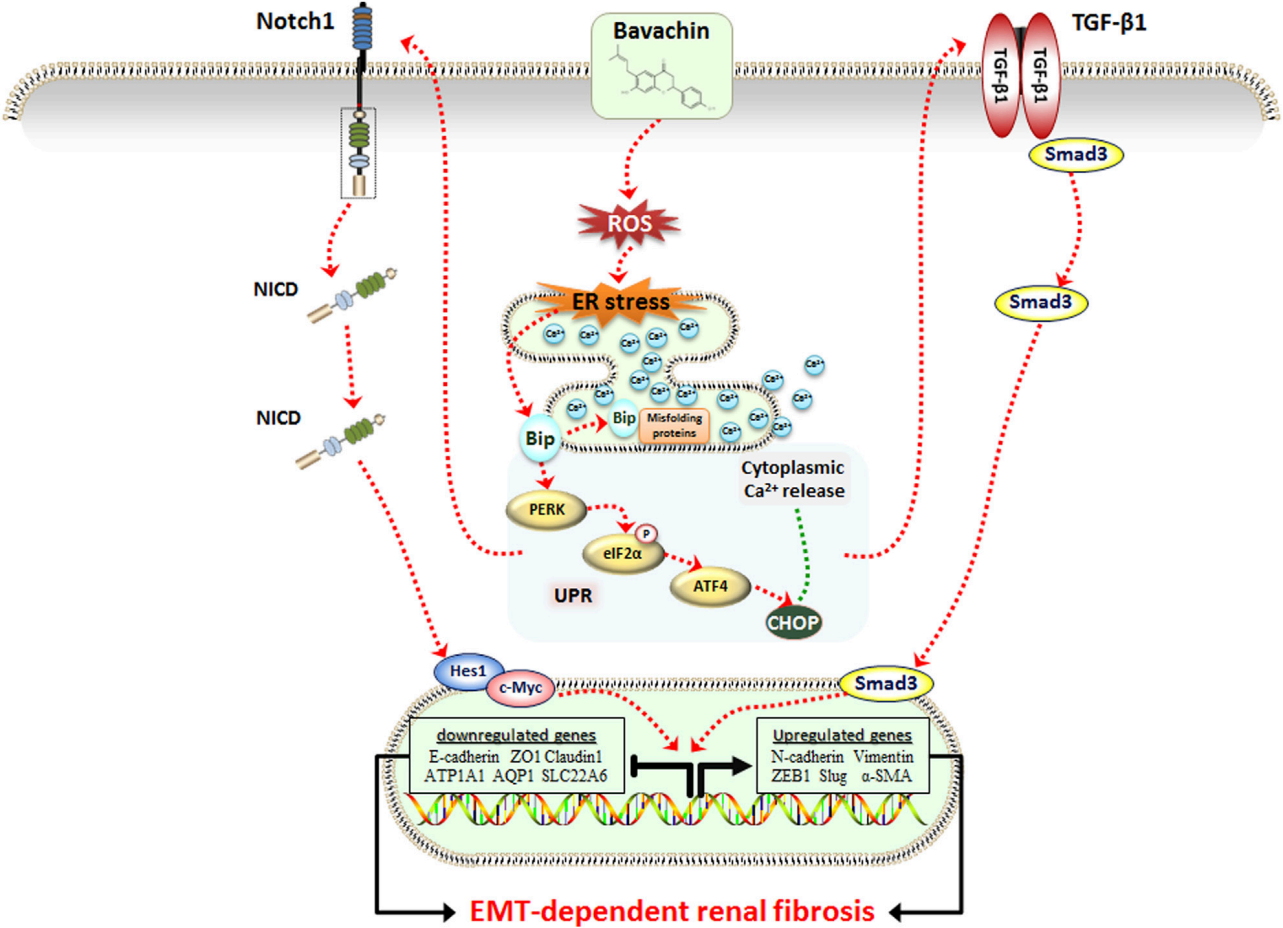
Schematic representation of the potential molecular mechanism underlying BV-induced renal fibrosis.

In summary, this study first confirmed that low dose of BV could cause obvious EMT-related renal fibrosis *in vivo* and *in vitro*, which was associated with the activation of the TGFβ1/Smad3 and Notch1/NICD signaling pathway. During this progress, elevated ER stress and cytoplasmic Ca^2+^ overload, both of which were evoked by the ROS-triggered Bip/eIF2a/CHOP signaling pathway, play critical roles in BV-induced renal fibrosis (Figure 6). Furthermore, we discovered for the first time that ginsenoside Rb1 exerted an outstanding effect against BV-induced renal fibrosis via suppressing ROS-triggered ER stress following EMT. Hence, this research helped to understand the nephrotoxicity caused by low-dose of BV and its toxic model of action and provided evidence that ER stress could be an alternative target and ginsenoside Rb1 could be a promising natural ingredient for the treatment of BV- or *Fructus Psoraleae*-induced renal fibrosis.

## Data Availability

The original contributions presented in the study are included in the article or Supplementary Material. Further inquiries can be directed to the corresponding authors.

## Abbreviations

BV, bavachin; EMT, epithelial–mesenchymal transition; ROS, reactive oxygen species; NAC, N-acetyl-L-cysteine; ER, endoplasmic reticulum; TCM, traditional Chinese medicine; CKD, chronic kidney disease; Ca^2+^, calcium ions; ERK, extracellular signal-regulated kinase; UPR, unfolded protein response; Ero1α, endoplasmic reticulum oxidoreductase-1; IP3R, inositol triphosphate receptor; TM, tunicamycin; HTS, high throughput screening; 4-PBA, sodium 4-phenylbutyrate.
